# Predictors of correct technique in patients using pressurized metered dose inhalers

**DOI:** 10.1186/s12890-017-0386-6

**Published:** 2017-02-28

**Authors:** Kyra Bartolo, Martin Balzan, Emma Louise Schembri, Rachelle Asciak, Darlene Mercieca Balbi, Michael Pace Bardon, Stephen Montefort

**Affiliations:** Mater Dei Hospital, Dun Karm Street, Msida, Malta

**Keywords:** Healthcare professional, Critical step, Instruction, Human error, Medicinal efficacy

## Abstract

**Background:**

﻿Corret inhaler technique is recommended by guidelines for optimum asthma care. ﻿The objective of the study is﻿ to determine real life predictors of correct pressurized metered dose inhaler (pMDI) technique in Asthma and COPD patients.

**Methods:**

Two hundred eight adult patients aged 18+ from respiratory outpatients (69.2%) and the community on regular pMDI for a diagnosis of Asthma (78.9%) or COPD, were recruited. A questionnaire containing 31 possible predictors was administered and pMDI technique with or without spacer was observed by trained researchers on 12 point steps, of which 4 were considered critical.

**Results:**

23.1% of patients had no errors in inhaler technique and 32.2% had no critical errors. Patients had a median of 10 correct steps (IQR9-11), and 3(IQR2-4) correct critical steps. Using binary logistic regression the predictors of 10 correct steps were, other healthcare professional (pharmacist, nurse, physiotherapist) explained OR 3.73(1.63–8.54, *p* = 0.001), male gender 2.70(1.35–5.39, *p* = 0.004), self-score 1–10 1.21(1.05–1.39, *p* = 0.007), spacer use 0.38(0.19–0.79, *p* = 0.007), inhaled steroid 3.71(1.34–10.25, *p* = 0.01), heart disease 0.31(0.13–0.77, *p* = 0.01), pneumococcal vaccine 2.48(1.0–6.15, *p* = 0.043), education level 1–4 1.44(1.00–2.06, *p* = 0.05) and respiratory physician explained 0–7 times, 1.11(0.99–1.26, *p* = 0.08). Using ordinal logistic regression, predictors for correct critical steps 0–4, were: technique self-score 1–10 1.2(1.05–1.42, *p* = 0.006), inhaled corticosteroid use 2.78(1.1–7.31, *p* = 0.03) and education level 1–4 1.41(1.02–1.95, *p* = 0.03 Times respiratory physician explained inhaler technique 0–7 1.1(0.98–1.24, *p* = 0.1), married status 1.55(0.85–2.82, *p* = 0.15), hypercholesterolaemia 0.52(0.25–1.01, *p* = 0.054) and male gender 1.76(0.97–3.18, *p* = 0.06).

**Conclusions:**

Known predictors of correct pMDI use, such as gender and education level were confirmed, while age and concomitant use of dry powder inhaler were not. Pneumococcal vaccination and awareness of steroid side effects were possible novel positive predictors, while the use of a spacer and co-morbidity with heart disease were found to be negative predictors. Patients’ self-assessment correlated well with actual performance. This information may be useful in defining approaches to optimize inhaler techniques which are so susceptible to human error.

**Electronic supplementary material:**

The online version of this article (doi:10.1186/s12890-017-0386-6) contains supplementary material, which is available to authorized users.

## Background

Guidelines for asthma and chronic obstructive pulmonary disease (COPD) recommend various treatment strategies, which include the use of inhaler devices, mainly pressurized metered dose inhalers (pMDI) with or without spacer devices, or dry powdered inhalers (DPI) [1–3]. Locally delivered small doses have the clear advantage over larger oral doses with respect to systemic side effects.

While guidelines are based on controlled studies on motivated patients, in real life it has been shown that a large proportion of patients, are unable to use their inhaler devices properly committing a number of often critical errors which might compromise the clinical efficacy [4–6]. Various studies have reported that only 10–50% of patients are able to use their inhalers without error [7]. Inadequate knowledge and training of patients, increasing age, female gender and low education [8] have been identified by some studies as possible causes of poor inhaler technique [9–11].

At least two studies have clearly shown that poor inhaler technique was associated with worsening respiratory symptoms [6, 7]. However this effect may be worsened or mitigated by other covariates, such as poor adherence to medication, smoking, the use of spacer devices or the actual stepping up of medication as recommended by guidelines.

Malta is an island in central Mediterranean with a population of 425,000 [12] with a high prevalence of asthma [13]. Separate studies from Malta reported proper inhaler technique in adults at 13.8% and 63.5% with 2 errors or more [14]. In another study, [15] 83% of children performed correctly two out of the 4 critical steps assessed. The aim of this study was to try to identify real life predictors of inadequate inhaler technique in Asthma and COPD patients, from a large array of possible predictors, which included aspects of asthma care and physician contact, demographic data, co-morbidities, vaccination history, sources of information and training of inhaler technique.

## Methods

Two hundred eight patients aged 18 and older were recruited from two main sources, the asthma and respiratory outpatient clinics of two respiratory consultants at Mater Dei Hospital, and community based patients from hospital records of patients who were known to receive prescriptions for regular inhaler medication. While hospital patients were invited to participate prior to their routine appointment while visiting hospital, the latter were contacted by phone by 3 of the authors and invited to attend for an outpatient visit at Mater Dei hospital. A face to face questionnaire was administered by one of six medical practitioners or two final year medical students, once formal consent was obtained in writing, prior to the hospital outpatient visit. All 8 researchers/assistants had received prior training of how to deliver the questionnaire but were not directly responsible for patient care. The questionnaire which had been validated by two peer reviewers prior to the start of data collection included various questions related to asthma care, demographic data, co-morbidities and vaccinations, sources of inhaler knowledge, physician contacts and possible respiratory outcomes. The questionnaire was carried out in the Maltese language, except for 15 English-speaking patients who used the English translation. This version had been verified by back translation.

The patient was than directly observed by the interviewer using the inhaler device. All interviewers received prior instruction and scored the performance on a check list. A 12 point checklist was used for metered dose inhaler with or without spacer use adapted from the American Thoracic Society [16], adding the manufacturers’ advice to keep the head straight. The choice of critical steps was a selection from those recommended by Newman [17]. The first assessments of technique by an interviewer were observed by one of the first two authors of the study. Table 1 shows the relative check list. Errors numbered 2, 6, 8 and 10 were considered to be critical errors.

**Table 1:** Inhaler Technique check-list

Step	pMDI + Spacer	pMDI
**1**	Remove the cap from the inhaler.	Remove the cap from the inhaler.
**2**	**Shake the inhaler well for 5s**	**Shake the inhaler well for 5s.**
**3**	Insert the inhaler into the open end of the chamber and ensure that the inhaler fits properly.	Hold the inhaler firmly by placing your index finger on top of the canister, and thumb on the bottom of the mouthpiece.
**4**	Sit up straight or stand up.	Sit up straight or stand up.
**5**	Tilt your head back slightly.	Tilt your head back slightly.
**6**	**Exhale completely away from the spacer.**	**Exhale completely away from the inhaler.**
**7**	Place the mouth piece in your mouth and seal your lips tightly around it.	Place the inhaler in your mouth and seal your lips tightly around it.
**8**	**Press the inhaler and breathe in steadily and deeply.**	**Press the inhaler and breathe in steadily and deeply.**
**9**	Remove spacer from the mouth.	Remove the inhaler from the mouth.
**10**	**Hold your breath for 10s or as long as is comfortable.**	**Hold your breath for 10s or as long as is comfortable.**
**11**	Exhale slowly.	Exhale slowly.
**12**	Remove the inhaler from the chamber and replace covers.	Replace cap on inhaler.

Critical steps are shown in bold

The study was approved by the Mater Dei Hospital Data Protection Committee on 26^th^ November 2014. Each participant gave written formal consent.

### Statistical methods

Two criteria for inhaler technique were tested. The first one was the comparison of the characteristics of patients who completed 10 correct steps out of a total of 12 (representing the median value of correct steps, 63.9% of patients) with those completing 9 or less, and a second criterion comparing patients with no critical errors out of a total of four (32.2%) with those having at least one critical error. The latter criterion isclearly more stringent and the desired optimal performance.

Three separate methods were used to statistically establish predictors of correct inhaler technique for all 12 steps and the 4 critical steps.

A univariate analysis of each of the 31 predictors comparing the characteristics of those with 10 or more correct steps out of 12 with those with < 10, and another comparing patients with no critical errors with patients with at least one critical error was performed. Comparison of proportions was carried out with Fisher test, while comparison of baseline characteristics with mean and standard deviation was performed using t tests. To determine independent odds ratios for the 31 predictors for a positive response to each of the two criteria two separate multivariate binary logistic regression models were used. A third model for predictors on no errors (0/12) was also tested. Predictors with a *p* value >0.15 were filtered and removed from the model using stepwise regression.

In an effort to eliminate the effect of previously untested cut-off points for the satisfactory number of correct steps another two models using ordinal logistic regression with all 31 predictors were used to determine odd ratios of predictors of the number of correct steps 0–12 and the number of critical steps 0–4 of inhaler technique. The correlation between self-assessment of technique 1–10 and the total number of correct steps 0–12 was tested using both Spearman and Pearson’s methods. Minitab 17 software was used

## Results

The patients included in this study were on regular treatment for asthma (79%) or COPD and were using a pMDI. The patient characteristics are shown in Table 2. Inhaled corticosteroid (87%) and/or inhaled ipratropium (18.3%) treatment was provided via pressurized metered dose inhaler. Long acting Beta agonists (32.7%) were delivered via formoterol aerolizer.

**Table 2:** Characteristics of patients using regular metered dose inhalers, and univariate comparison of 10 correct steps vs 3 errors or more, and no critical error vs critical errors

	All	10–12 steps	<10steps	*P* value	No critical error	critical error	*P* value
All	208	133(63.9%)	75(36.1%)		67(32.2%)	141(67.8%)	
**Male gender**	**97(46.6%)**	**69(51.9%)**	**28(37.7%)**	**0.04**	31(46.3%)	66(46.8%)	0.94
Mean age(STD)	57.6(15.4)	57(14.5)	58.6(16.9)	0.45	57.6(12.5)	57.6(16.6)	0.99
**Asthma diagnosis**	**164(78.9%)**	**110(82.7%)**	**54(72.0%)**	**0.08**	57(85.1%)	107(75.9%)	0.11
Advised to use spacer	170(81.7%)	109(82%)	61(81.3%)	0.91	56(83.6%)	114(80.9%)	0.626
**Observed to use spacer**	**132(63.5%)**	**78(58.6%)**	**54(72%)**	**0.05**	38(56.7%)	94(66.7%)	0.17
**Inhaled corticosteroids**	**180(86.6%)**	**120(90.2%)**	**60(80%)**	**0.05**	59(88.1%)	121(85.8%)	0.65
LABA via aerolizer	68(32.7%)	43(32.3%)	25(33.3%)	0.88	19(28.4%)	49(34.8%)	0.35
Inhaled ipratropium bromide	38(18.3%)	21(15.8%)	17(22.7%)	0.24	30(21.3%)	8(11.9%)	0.12
**Mean education 1–4 (STD)**	**2.09(0.98)**	**2.21(1.03)**	**1.88(0.85)**	**0.014**	2.24(1.0)	2.02(0.97)	0.14
Married status	136(65.4%)	92(69.2%)	44(58.7%)	0.13	48(71.6%)	88(62.4%)	0.18
Smoker Y/N	24(11.5%)	13(9.8%)	11(14.7%)	0.31	7(10.4%)	17(12.1%)	0.72
Resp physician Follow up	144(69.2%)	92(69.2%)	52(69.3%)	0.98	43(64.2%)	101(71.6%)	0.26
**Resp physician explained Y/N**	185(88.9%)	118(88.7%)	67(89.3%)	0.89	63(94%)	**122(86.5%)**	**0.06**
Resp explained – number of times 1–7	3.37(2.8)	4(2.70)	3.37(2.62)	0.16	3.9(2.64)	3.74(2.71)	0.28
Resp physician time to explain 1–5	1.81(1.37)	1.91(1.46)	1.63(1.18)	0.13	1.85(1.38)	1.78(1.37)	0.76
GP follow up	87(41.8%)	54(40.6%)	33(44%)	0.94	27(40.3%)	60(42.6%)	0.8
GP explained Y/N	104(50%)	69(51.9%)	35(46.7%)	0.47	32(47.8%)	72(51.1%	0.66
GP explained times 0–7	1.43(2.19)	1.40(2.12)	1.48(3.27)	0.8	1.36(2.12)	1.46(2.22)	0.75
GP time to explain(0–5)	0.97(1.25)	1(1.27)	0.92(1.23)	0.66	0.96(1.33)	0.98(1.22)	0.9
Any follow up	187(89.9%)	119(89.5%)	68(90.7%)	0.78	59(88.1%)	128(90.8%)	0.56
**Other health care professional explained**	**60(28.9%)**	**48(36.1%)**	**12(16%)**	**0.001**	21(31.3%)	39(27.7%)	0.59
Info-other source	86(41.4%)	56(42.1%)	30(40%)	0.77	31(46.3%)	55(39%)	0.37
**Years of inhaler use (decades)**	**1.88(1.59)**	**2.02(1.58)**	**1.62(1.58)**	**0.08**	2.01(1.46)	1.82(1.65)	0.4
**Technique self-score 0–10**	**7.8(2.25)**	**8.06(2.02)**	**7.28(2.56)**	**0.025**	7.96(2.21)	7.7(2.28)	0.434
Perception as effective 1–5	2.45(0.8)	2.4(0.735)	2.48(0.84)	0.47	2.4(0.74)	2.48	0.47
Ease of use 1–5 (SD)	3.1(1.0)	3.15(0.97)	2.97(0.99)	0.21	3.12(0.95)	3.07(1.0)	0.74
**Concern inhaler side effects 0/1**	44(21.2%)	30(22.6%)	14(18.7%)	0.6	**19(28.4%)**	**25(17.7%)**	**0.09**
Diabetes	34(16.35%)	20(15%)	14(18.7%)	0.51	12(17.9%)	22(15.6%)	0.68
Cholesterol	59(28.4%)	33(24.8%)	26(34.7%)	0.14	16(23.9%)	43(30.5%)	0.31
Hypertension	93(44.71%)	56(42.1%)	37(49.3%)	0.32	30(44.8%)	63(44.7%)	0.99
**Heart disease**	**34(16.4%)**	**14(10.5%)**	**20(26.7%)**	**0.005**	**7(10.4%)**	**27(19.1%)**	**0.08**
Influenza vaccine (current)	121(58.2%)	44(57.9%)	77(58.7%)	0.91	36(53.7%)	85(60.3%)	0.38
**Pneumococcal vaccine**	**40(19.23%)**	**30(22.6%)**	**10(13.3%)**	**0.08**	13(19.4%)	27(19.1%)	0.97

(*p* <0.1 shown in bold)

**Fig. 1:**
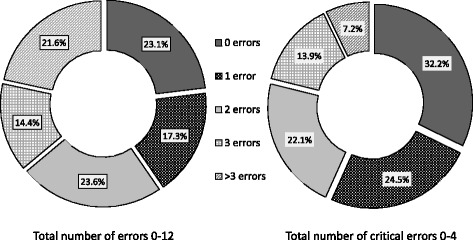
Percentage of patients having errors in pMDI inhaler technique. (*n* = 208)

Despite the fact that 89.9% of these patients had regular follow up with either a respiratory physician (69.2%) or a general practitioner (41.8%), only 23% of patients had no errors in inhaler technique and 32.2% no critical errors as demonstrated in Fig. 1

**Fig. 2:**
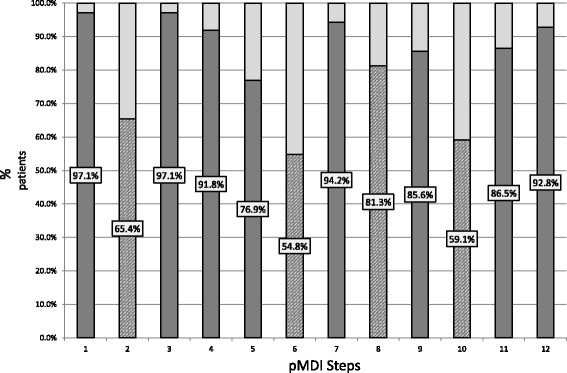
Percentage number of patients with correct steps per step (*n* = 208). (Lower half of column indicates percentage participants performing step correctly, dark grey for non-critical steps, diagonal shading indicates critical steps)

The 3 steps with the highest error rate were all critical steps, Step 2 (34.6%) ‘shake the inhaler well for 5 s’, step 6 (46.2%) ‘exhale completely away from the inhaler/spacer’ and step 10 (40.9%) ‘hold your breath for 10 s or as long as is comfortable”, as seen in Fig. 2.

**Fig. 3:**
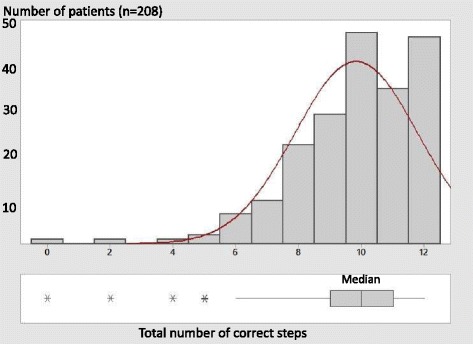
Number of correct steps of inhaler technique. (Median and interquartile range shown at the bottom. Asterisks are outlier values)

A respiratory physician had explained the technique to 89.9% of patients a mean of 3.37 (SD2.7) times, a general practitioner to 50% of patients 1.43 (SD2.2) times. Only 28.9% had received advice from another health care professional, while 41.4% had sought other sources of information in the written (34%) or electronic media (23%). Figure 3 shows the distribution of correct number of steps. However the median of 10(IQR9-11) correct steps out of 12, and 3(IQR2-4) out of 4 correct critical steps indicates that most patients were getting at least 9 steps right.

On univariate analysis, male gender (*p* = 0.04),, higher level of education on a 1–4 scale (*p* = 0.014), patient self-rating of technique on a scale of 1–10 (*p* = 0.025), use of inhaled corticosteroids (*p* = 0.05), advice by other health care professional (*p* = 0.001) were higher in patients with 10 or more good steps. On the other handuse of spacer (*p* = 0.05) and the presence of heart disease (*p* = 0.005) were less frequent in this group. Instruction of inhaler technique by respiratory physicians (*p* = 0.98) or by general practitioners (*p* = 0.94) did not have a significant effect on the performance of 10 or more correct steps.

On the other hand, none of the personal characteristics above were more frequent amongst patients with no critical errors. Table 3 shows the results of binary logistic regression and stepwise selection predicting either 10 good steps, no critical error and no errors at all. It established 8 predictors for the ability to perform 10 steps; odd ratios of predictors of 10 good steps were highest for explanation by other health care professional OR3.73 (1.63–8.54, *p* = 0.001), male gender 2.70 (1.35–5.39, *p* = 0.004), patient education level 1.44 (1.0–2.06, *p* = 0.045) self-rating score for inhaler use 1.21 (1.05–1.39, *p* = 0.007), use of inhaled corticosteroid 3.71 (1.34–10.25, *p* = 0.01), and pneumococcal vaccination 2.48(1.00–6.15, *p* = 0.043). Usage of a spacer device 0.38(0.19–0.79, *p* = 0.007) and heart disease 0.31(0.13–0.77, *p* = 0.011) were negative predictors As regards to predictors for no critical errors, none of the 31 reached statistical significance. While only explanation by a respiratory physician predicted absolutely zero error. Table 4 shows a separate analysis using ordinal logistic regression which confirmed that besides the same predictors of 10 positive steps, another predictor for a correct step was explanation by the GP OR 3.3(1.35–8.09, *p* = 0.009). Predictors for correct critical steps, were technique self-score 1.2(1.05–1.36, *p* = 0.006), inhaled corticosteroid 2.78(1.1–7.02, *p* = 0.03) and education level 1.41(1.02–1.95, *p* = 0.036). 4 predictors just failed to reach statistical significance, male gender 1.76(0.97–3.18, *p* = 0.06), times respiratory physician explained inhaler technique 1.1(0.98–1.24, *p* = 0.1), married status 1.55(0.85–2.82, *p* = 0.15) andhypercholesterolaemia 0.52 (0.26–1.01, *p* = 0.054). Concomitant use of aerolizer was not an independent predictor for correct number of steps OR 0.7(0.4–1.26, *p* = 0.239) and OR0.81(0.45–1.47, *p* = 0.5) for critical steps.

**Table 3:**
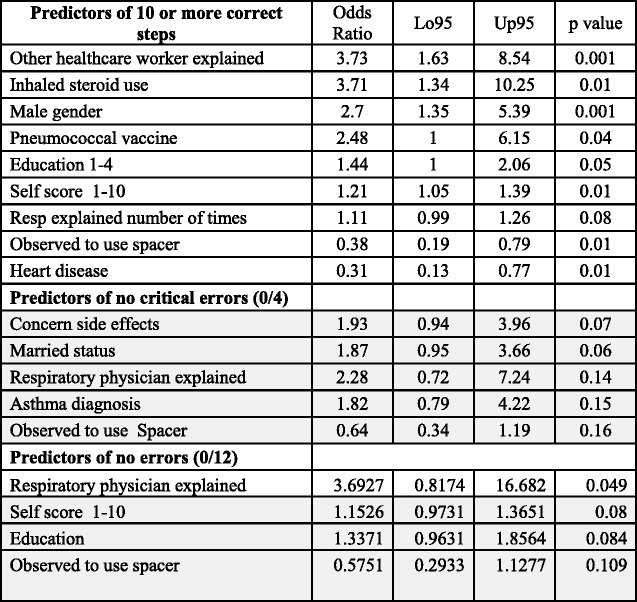
Odd ratios for predictors of inhaler technique by binary logistic regression and stepwise selection

(grey numbers *p* > 0.05)

**Table 4:** Predictors of correct steps of inhaler technique using ordinal logistic regression for all steps 0–12 and critical steps 0–4

Predictor of correct steps (0–12)	Odds ratio	Lo95	Up95	*p* value
Self score 1–10	1.25	1.1	1.42	0.001
Education 1–4	1.6	1.16	2.2	0.004
Observed to use spacer	0.43	0.24	0.8	0.008
GP explained	3.3	1.35	8.09	0.009
Male gender	2.07	1.15	3.71	0.015
Pneumococcal vaccine	2.36	1.14	4.86	0.02
Heart disease	0.43	0.2	0.9	0.026
Inhaled steroid use	2.45	0.98	6.1	0.054
Resp explained number of times	1.11	0.99	1.25	0.068
Any follow up	0.38	0.12	1.22	0.104
High cholesterol	0.58	0.3	1.12	0.104
Times explained by GP	0.88	0.74	1.04	0.126
Other healthcare-explained	1.55	0.86	2.8	0.147
Predictor of correct critical steps	Odds Ratio	Lo95	Up95	*p* value
Self score 1–10	1.2	1.05	1.36	0.006
Inhaled steroid use	2.78	1.1	7.02	0.031
Education 1–4	1.41	1.02	1.95	0.036
High cholesterol	0.52	0.26	1.01	0.054
Male gender	1.76	0.97	3.18	0.063
Resp explained number of times	1.1	0.98	1.24	0.101
Observed to use spacer	0.62	0.33	1.15	0.129
Married status	1.55	0.85	2.82	0.15

Pearson correlation between self-score and total number of correct steps was 0.224 (*p* = 0.001) and spearman was 0.184 (*p* = 0.008).

## Discussion

The study population represented a number of patients on regular inhaled treatment which were mixed between hospital outpatients, and the general community with only 11% without regular follow-up. In Malta, asthma medication is prescribed free of charge however choice of medication is limited to the government formulary. Inhaled corticosteroid and ipratropium during the study period were available via pmdi with or without a spacer device while LABAs were provided via aerolizer. All patients had received instruction on use, mainly from the respiratory physician, and/or the general practitioner or other health care professional. Despite this only 23.1% performed all 12 steps correctly, and 32.2% all 4 critical steps correctly, though most patients had 9 or more correct steps as seen in Fig. 3. This is slightly higher from previous Maltese data which was only hospital based [14] but consistent with higher values of international data [6, 7, 9, 18–20].

Unfortunately the list of correct steps varies greatly between studies, the most recent being from the American Thoracic Society [16], from which our criteria have been adapted together with the manufacturers’ advice to keep the head straight. The choice of critical steps was a selection from those recommended by Newman [17].

Three different statistical analyses were carried out, but while the results were slightly different, they were consistent, with the ordinal logistic regression model having produced the strongest statistical significance., As interdependent variables could have have affected the mathematical models differently, resulting in variations in the odds ratios and *p* values between models all results are presented, rather than selecting only those with the strongest statistical significance. Analysis of completion of all 12 steps by binary logistic regression resulted in only one predictor, probably because only a few patients managed achieve this goal.

As 90% of subjects had received a demonstration by a respiratory physician, the main predictor appeared to be the number of demonstrations, while as fewer patients had demonstrations by family doctors or other health care professionals the statistical effect of that benefit was clearer. However an explanation by a respiratory physician was the only predictor for no errors at all. There seemed to be no benefit in this group for other information from written or electronic media.

Of interest is how instruction by other health care professional and general practitioner was a predictor of performing the 10 steps correctly. On the contrary, critical steps were better predicted by respiratory physician intervention, in particular the number of times explained. From univariate analysis it appeared that in the local context respiratory physicians are spending nearly twice as much time to explain than general practitioners.

As most had regular physician contact, most predictors were individual characteristics. The best predictor in fact was the patient’s own evaluation as had been suggested by Erikson [18], and the patient’s level of education, a consistent predictor in a number of studies [9].

Male gender was a stronger predictor of the 10 correct of steps, and weaker for critical steps. Men have been reported to have better hand to eye coordination [21] and spatial three dimensional ability than women [22]. On the other hand, a possible reason why the effect is weaker with critical steps might be that the accuracy and performance of critical steps depends on a sound understanding of the process and one can speculate that it is the result of a personal motivation to look after one’s health which may beseparate from simple co-ordination [23]. When considering hand to eye coordination and surgical skills amongst surgical trainees Ali et al reported that without instructor feedback, males outperform females, whereas females and males performed equally with instructor feedback [21]. Furthermore after correction for computer game participation there seemed to be no difference between genders [21].

Patients with asthma receiving inhaled corticosteroids were also more likely to perform more correct steps. This effect cannot be attributed to age but one possible reason, is the strong element of reversibility of airway obstruction when compared to COPD which could re-inforce motivation to use the inhaler properly. On the other hand COPD patients may have constant exercise limitation and difficulty with breathing affecting inhaler technique as suggested by Al-showair [24].

While 81.7% of patients were prescribed a spacer, only 63.5% were using it. This was already noted in the local context [14]. Spacer use was a negative predictor for both correct steps, and correct critical steps. One can speculate a number of possible explanations in that doctors were more likely to prescribe spacers to patients with poor technique as is recommended by guidelines, or else patients with poor technique because of symptoms were more likely to use it. Furthermore because of its cumbersome size patients with good technique and few symptoms might have actually decided not to use it, or else the spacer itself might have lulled some patients into a false sense of security who might become complacent with the accuracy of technique.

Fear of side effects of inhalers was another possible predictor which perhaps might be leading to patients being more careful with inhalers and avoiding critical errors. While it has been suggested that perceptions and misconceptions may have an effect on adherence to medication [25], we were unable to find any study on the effect on inhaler technique.

Contrary to other studies [9], concomitant use of another device, in this case the aerolizer, did not have a statistically significant effect even though the aerolizer requires a short sharp inhalation, which is quite different to pMDI technique. However one must note that step 8 had an error rate of 18.7%. Perhaps as it is just one of 12 steps, the number of patients was not large enough to detect the effect.

During the study period, pneumococcal vaccine was not free of charge and had to be purchased, and this could explain that as a predictor it probably reflected a stronger motivation to look after oneself [23]. On the other hand the influenza vaccine is provided free of charge, and perhaps that could explain why it was not as good a proxy predictor for motivation.

The occurrence of heart disease and possibly hypercholesterolaemia as negative predictors of inhaler technique has not been previously reported. A reference to this effect could not be located and perhaps this might be something particular to the small sample studied. However one can speculate two possible reasons, one of which is that in cardiac disease, treatment is by one day curative procedures such as dilatation with stent insertion, and prevention by oral drugs leading patients to be unaccustomed or complacent about the critical importance of inhaler technique. Another reason could be that cardiac patients might be less keen on the use of inhalers because of their possible cardiac side effects such as palpitations or else attributing their symptoms to cardiac rather than respiratory disease.

Married status seems to be a possible positive predictor of performing critical steps correctly, just failing to reach statistical significance. While partner support in chronic disease is clearly beneficial [26] the benefit of training the partner as an independent observer on inhaler technique to date has not been studied or documented.

The strengths of this study were that there was a mix of hospital based patients seen regularly by respiratory physicians and community patients seen mainly by general practitioners all using a limited set of medications and all receiving a pMDI, allowing focus on the cheapest and most widely used mode of treatment. The extensive number of predictors tested, and the high number of total or critical steps and the fact that the steps were based on evidence from scientific literature allowed a broad analysis of pMDI technique. The main weakness was that inhaler technique was assessed by human observers rather than video camera, or flow instruments possibly leading to possible inaccurate assessment and inter observer bias. Community patients selected on the basis of records of regular use of medication who refused the invitation might have been one side poorly motivated or otherwise well controlled due to optimal technique leading to a degree of selection bias. The number of COPD patients was small and under-represented. Furthermore the number of patients receiving aerolizer was insufficient to allow a separate evaluation.

Despite the small sample size, a Signiant number of predictors were detected. This reinforces the view that these predictors, have a strong clinical effect which is easily measurable. It is possible that a larger sample could have detected more predictors which possibly have lesser impact. However while the results are similar to other studies they are not necessarily totally generalizable to other health care systems or different populations with different cultures and attitudes towards disease. In a scenario where patients are seen frequently and their technique routinely reviewed what are the options to improve technique? This has been addressed by Price et al on behalf of the “Inhaler error steering committee” [5]. Many times, errors might arise because many health care professionals providing instruction are not aware themselves of all the correct steps and possible mistakes. This is the likely explanation as to why the respiratory physician could have had greater impact on critical steps. This can be addressed by specific training courses for healthcare professionals on whom most patients rely for information rather than printed or electronic media. While patient knowledge and competence also needs to be addressed, the mode of instruction may also need to be clearly communicated to the less educated patients with visual demonstration in real life or video demonstration [27] and perhaps allocating more time. While one study failed to show any benefit in technique from increased specific knowledge [9], in the experience of the authors, patients who understand the mechanics of inhaler technique are more likely to learn.

Studies on whether patients would benefit from a switch to dry powder inhaler have been conflicting [4]. Indeed an inhaler switch without consultation was associated with worsened asthma control however there is evidence that shows that a patient is more likely to use an inhaler he or she prefers [4, 28]. This individual approach is supported by BTS guidelines [1]. The option of providing a spacer does contribute to better bioavailability even with imperfect technique and is recommended for patients with poor co-ordination, besides those on high dose inhaler steroids so as to reduce throat deposition [29]. Chapman et al have recommended a specific algorithm for the choice of inhaler [30].

Finally perhaps there is room for improved technology, perhaps with new inhalers with smaller particle size, and simpler use and design which could make the airway delivery of medicinals by inhaler devices more reliable and less operator dependent [5, 28, 31].

## Conclusion

This real life study showed that in this group of patients treated with pMDI, with regular follow up and instruction, while most achieved nine out of 12 correct steps, only 23% had no errors in technique. While previously established predictors like gender, patient education and instruction by health care professional were confirmed, the effect of age and negative effect of use of different inhaler devices was not. This study also provides novel information on predictors of inhaler technique. Seeking pneumococcal vaccination and awareness of steroid side effects predicted fewer critical errors, possibly reflecting a motivation to look after oneself [23]. Use of a spacer was a negative predictor of good technique, possibly because doctors are more likely to prescribe it in patients with poor technique, and patients are more likely to utilize it when they have insight into their short-comings. In fact, patient self-assessment was statistically correlated with actual performance. Surprisingly co-morbidity with heart disease was a negative predictor possibly reflecting a dependence on prevention in tablet form and single procedure cures.

Intense follow-up of patients failed to produce optimal results, indicating that most predictors of good pMDI technique are patient dependent possibly reflecting attitudes which may be modified. Two different strategies may be possible to achieve better results in inhaler technique. One can either attempt to optimize and individualize the educational intervention on patients, or rely on technological advances of inhaler devices making them easier to use and more forgiving of human error.

## Additional files

Additional file 1: Dataset. (XLSX 56 kb)
Additional file 2: Questionnaire. (PDF 157 kb)

## Abbreviations

COPD: Chronic obstructive pulmonary disease
DPI: Dry powder inhaler
PMDI: Pressurized metered dose inhaler
