# Supplementary material for: Predictors of correct technique in patients using pressurized metered dose inhalers
Source: BMC Pulm Med. 2017 Feb 28;17:47. doi: 10.1186/s12890-017-0386-6 (PMC5331728; doi:10.1186/s12890-017-0386-6)
Supplement: Additional file 2: — Questionnaire. (PDF 157 kb) [file 12890_2017_386_MOESM2_ESM.pdf]

Predictors of Inhaler Technique

To be filled in by patient:

Name: ______________________ **Surname:** ______________________

Age: _______________ **Gender:** M F

Locality: ____________________ **Status:** Single Married Divorced Widow/er

Education: Primary Secondary Sixth Form University

Employment: Student Employed Unemployed Retired Housewife

Contact no: _________________________

Do you suffer from: Asthma COPD

At what age did you start using inhalers? _______________years old

Who diagnosed your lung condition: (choose one) Pulmonary physician GP

Who follows you up regularly for this condition? Pulmonary physician GP Both

How often? 0 – 4 months 4 – 8 months 8 – 12 months >12 months

Has your pulmonary physician ever explained the inhaler technique?

Yes No

Number of times 1 2 3 4 5 >5 At every outpatient visit

For how long 2 4 6 8 10 minutes

Has your GP ever explained the inhaler technique?

Yes No

Number of times 1 2 3 4 5 >5 At every visit

For how long 2 4 6 8 10 minutes

Has any other healthcare worker ever explained the inhaler technique?

No Yes **Who?** Nurse Physiotherapist Pharmacist

For how long? 2 4 6 8 10 minutes

During the last 4 weeks how often did you use the Salbutamol inhaler (Ventolin)?

Everyday 4-5 times a week 2-3 times a week once or less

During the past year how many times did you require steroids because of shortness of breath / cough / sputum?

_______________ Times

During the past year how many times did you need admission to hospital because of your lung condition? _____ Times

During the past year how many times did you need to go to hospital or health centre for nebulisers?

_______________times

Did you ever need ITU admission because of your lung condition? Yes _______ times No

Do you currently smoke tobacco: on a daily basis less than daily not at all

How many cigarettes do you smoke on a daily basis: 0-5 5-10 10-20 20-40 >40

For how many years have you smoked: 0-5 5-10 10-20 20-40 >40 years

If you currently do not smoke, have you ever smoked Yes No

If yes: on a daily basis less than daily

How many cigarettes did you smoke on a daily basis: 0-5 5-10 10-20 20-40 >40

For how many years did you smoke: 0-5 5-10 10-20 20-40 >40 years

At what age did you quit: _______________

Do you suffer from allergic rhinitis? Yes No

Have you ever had allergy skin tests done? Yes No

Have you ever had allergy blood tests done? Yes No

What did the results show? Yes I am allergic to ____________________ I do not have any allergies

Do you suffer from any other co-morbidities?

Hypertension Hyperlipidaemia Diabetes Heart disease Stroke

Other: ____

Do you take the influenza vaccine every year? Yes No **In the last year?** Yes No

Did you ever take the Pneumococcal vaccine? Yes No

Have you ever read/watched the inhaler technique explanation on:

Leaflet Book Magazine Television Internet

From 1 to 10 how would you grade your inhaler technique (10 being the best)? _______________

How do you rate the use of inhalers? Very easy Easy Normal Difficult Very difficult

How beneficial is the inhaler on your breathing? Very effective Effective Not effective

Do you have any concerns regarding possible side effects of your treatment? Yes No

If yes, which ones: ____

Do you rinse your mouth after using the yellow/brown/orange inhaler? Yes No NA

Does any other person in your household use inhalers? Yes No

To be filled in by doctor/medical student:

What inhalers does the patient use? pMDI Aeroliser

Does the patient use a spacer? Yes No

Score for inhaler technique:

pMDI + spacer /12 pMDI /12 Aeroliser /14

Last FEV1:
